# Assessment of patient compliance in orthokeratology and analysis of influencing factors: a cross-sectional study

**DOI:** 10.1186/s12886-021-02148-2

**Published:** 2021-11-16

**Authors:** Zhiwen Bian, Xindi Xu, Duya Chen, Hailong Ni

**Affiliations:** The Second Affiliated Hospital, Zhejiang University School of Medicine, Eye Center, 88 Jiefang Road, Shangcheng District, Hangzhou, 310009 Zhejiang China

**Keywords:** Orthokeratology, Compliance, Influencing factors, Safety

## Abstract

**Background:**

Patient non-compliance, that is, failure to perform standard wear and care orthokeratology (ortho-k) lenses procedures, has been shown to be a major risk factor for contact lens-associated complications. Therefore, this study aimed to investigate the compliance with wear and care behaviors of ortho-k patients and analyze its influencing factors.

**Methods:**

Patients who were successfully prescribed ortho-k lenses at the Eye Center of the Second Affiliated Hospital of Zhejiang University School of Medicine (ECSAHZU) were enrolled in the study. Patient compliance with wear and care behaviors was examined through a questionnaire.

**Results:**

This study assessed 238 subjects. The subjects’ ages ranged from 7 to 25 (mean ± SD, 11.3 ± 2.5) years. The compliance with wear and care behaviors was 19.7%, and the subjects’ self-assessment compliance was 96.6%. The compliance rate of subjects wearing lenses for less than 1 year was higher than that of subjects wearing lenses for more than 1 year (*p* < 0.001). In the first year, the compliance rates of wearing experiences for less than 1 month, 1 month, 3 months, 6 months, and more than 6 months were 45, 29, 21.6, 20, and 27.6%, respectively, and there were no statistically significant differences in compliance among these periods (*p* = 0.314). No correlation was identified between compliance and age (*r* = − 0.061, *p* = 0.527) or sex (*r* = 0.114, *p* = 0. 751). There was no correlation between compliance and lens care operator (*r* = − 0.626, *p* = 0.151).

**Conclusions:**

The compliance of ortho-k patients was poor. After wearing ortho-k lenses for more than 1 year, compliance with wear and care behaviors declined. In clinical practice, measures should be taken to solve these problems and improve the safety of wearing ortho-k lenses.

**Supplementary Information:**

The online version contains supplementary material available at 10.1186/s12886-021-02148-2.

## Background

Myopia has become a global problem, with a recent study suggesting that close to 50% of the world’s population may be myopic, and as much as 10% may be high myopia [1]. High myopia can cause pathological ocular changes such as glaucoma, retinal detachment, and myopic macular degeneration, all of which can lead to uncorrected vision loss or even blindness [2–4]. Orthokeratology (ortho-k) is a type of specially designed rigid gas-permeable (RGP) contact lenses, which can reshape the cornea to temporarily reduce or eliminate myopia. Because ortho-k has emerged as one of the most effective methods to slow myopia progression in children [5–7] and has other advantages such as improving the uncorrected visual acuity during the daytime, there is a huge number of people who wear ortho-k lenses [8]. Although ortho-k has been proven safe for ocular tissues [9], because ortho-k is a contact lens, it can cause a series of contact lens-associated adverse events, including microbial keratitis, which can cause blindness [10, 11]. Patient non-compliance, that is, failure to perform standard wear and care ortho-k lenses procedures, has been shown to be a major risk factor for contact lens-associated complications [12, 13].

Sapkota K reported that 35.9% of medical doctors had a good level of compliance in conventional contact lenses (i.e., not including ortho-k lens) (*n* = 39) [14]. Robertson DM. et al. found that only 0.4% of patients were fully compliant with contact lens wear and care practices (*n* = 281) [15]. A multinational study by Morgan PB et al. showed that about 15% of daily disposable wearers were fully compliant, compared to close to 0 % for all other contact lens types (*n* = 4021) [16]. Cho P et al. found that the high-level compliance of ortho-k lenses, disinfection solutions, and lens accessories were 52, 54, 33%, respectively (*n* = 52) [17]. A study by Jiang et al. showed that the compliance rate of ortho-k was low, especially for wear and care behaviors (18.5%) (*n* = 405) [18]. Previous studies showed that the compliance of contact lens wearers was poor. Regarding the reasons for poor compliance, a study by Claydon [19] found that the contact lens patients were not intentionally non-compliant, but that non-compliance was the result of misunderstandings, forgetting, or poor guidance; only a small portion of non-compliant behaviors were intentional, with reasons such as inconvenience, neglect, or denial of risk. However, Claydon’s study did not include ortho-k patients. Ortho-k lenses are different from the RGP or soft contact lens that Claydon studied [19], and a substantial number of wearers of ortho-k lenses are adolescents [18, 20]; therefore, it is unclear whether the reasons for non-compliance are the same for ortho-k lenses. In addition, it is unclear when the compliance rate for wear and care behaviors dropped. Answering these questions can help eye care professionals (ECPs) take corresponding measures to improve the compliance rate for wear and care behaviors, which will enhance the safety of wearing ortho-k lenses. Therefore, this study aimed to determine the influencing factors behind non-compliance in patients prescribed ortho-k.

## Methods

The survey used a questionnaire developed by Jiang et al. [18] (Additional file 1). The following information was collected by questionnaire: patient demographic information, the independence of wear and care of the lenses, wear and care behaviors specified in the standard procedures and a self-assessment of compliance (i.e., whether patients believe his or her wear and care behaviors conform to the standard procedures). The wear and care behaviors included handwashing methods before handling lenses, lens cleaning procedures, use of expired solutions, procedures for soaking lenses, the interval of lens case replacement, exposure to non-sterile solutions, and the interval of lens deposition removal (Table 1).

**Table 1:** Compliance behaviors and compliance rates for orthokeratology patients with different wearing experience

Compliance behaviors	Compliance for group I (%) (***n*** = 156)	Compliance for group II (%) (***n*** = 82)	P^*^
1 Adequate hand washing	80.8	73.2	0.178
Washing hands before wearing lenses	98.1	97.6	
Washing hands before removing lenses	92.9	80.5	
Washing hands with soap	85.3	87.8	
2 Adequate lens cleaning	85.9	78.0	0.124
Cleaning lenses before wearing	98.1	95.1	–
Cleaning lenses after wearing	90.4	85.4	–
Rubbing and rinsing lenses	94.2	87.8	–
3 No use of expired solution	94.9	89.0	0.096
4 No topping off solution	92.3	95.1	0.41
Replacing total volume of solution with fresh solution	98.7	98.8	–
Replacing solution after each use	92.3	95.1	–
5 Lens case replacement according to recommendation of eye care professionals	55.8	24.4	< 0.001
6 No exposure to non-sterile solution	71.8	28.0	< 0.001
Drying hands after washing with tap water	76.9	47.6	–
No use of non-sterile solution when washing lenses	94.2	61.0	–
7 Removal of lens deposition interval according to recommendation of eye care professionals	67.3	39.0	< 0.001
**Compliance (%)**	26.9	6.1	< 0.001

Group I = subjects wearing orthokeratology lenses for less than 1 year. Group II = subjects wearing orthokeratology lenses for more than 1 year

^*^Chi-square test (χ^2^)

This study is a cross-sectional study. All patients who came to the Eye Center of the Second Affiliated Hospital of Zhejiang University School of Medicine (ECSAHZU) for follow-up were interviewed in June and July 2020. To ensure subjects were familiar with their wear and care procedures, patients who have ceased wearing ortho-k lenses were excluded.

The questionnaire was answered in the form of face-to-face interviews when ortho-k patients came to the hospital for follow-up. All subjects/subjects’ guardians gave informed written consent to participate in the study. Approval was obtained from all participants. This study followed the principles of the Declaration of Helsinki, ethical approval was obtained from the Ethics Committee of The Second Affiliated Hospital of Zhejiang University School of Medicine, and the ethical approval number of this study is IR2020001221.

The study used the Pearson chi-square test (χ^2^) to analyze compliance differences between the two groups, and multiple comparisons among the groups were also assessed using the chi-square test. In addition, multiple logistic regression was used to analyze the influencing factors of compliance, including age, sex, wearing experience, and lens care operator. Statistical analysis was performed using SPSS Version 23.0 (IBM Inc., Armonk, NY) software. A *p*-value of less than 0.05 was considered significant.

## Results

In total, 608 patients were interviewed, and 238 patients responded. The subjects’ ages ranged from 7 to 25 (mean ± SD, 11.3 ± 2.5) years, and 97.5% of subjects were younger than 18 years. The number of female and male subjects was 137 and 101, respectively. The subjects’ self-assessment compliance for wear and care behaviors was 96.6%, but the actual compliance for wear and care behaviors was 19.7%.

Results of the multiple logistic regression showed no correlation between compliance and age (*r* = − 0.061, *p* = 0.527) or between compliance and sex (*r* = 0.114, *p* = 0.751) (Table 2). Since most of the subjects were minors, lens care may be managed by their guardians as well as themselves. The impact of lens care operator had been considered. Multiple logistic regression showed that there was no correlation between compliance and lens care operator (*r* = − 0.626, *p* = 0.151) (Table 2).

**Table 2:** Analysis of influencing factors of compliance

	Wearing experience	Sex	Age	Care operator
***r***	−0.09	0.114	−0.061	−0.626
***p***^†^	0.001	0.751	0.527	0.151

^†^Multiple logistic regression

Multiple logistic regression indicated that there was a correlation between compliance and wearing experience (*r* = − 0.09, *p* = 0.001) (Table 2). A study by Jiang et al. showed that there was no correlation between wearing experience and compliance of patients who had worn ortho-k lenses for more than 1 year [18]. In order to investigate whether the compliance for wear and care behaviors decreased in the first year of wearing ortho-k lenses, subjects were divided into two groups according to the number of years of wearing experience: group I had been wearing ortho-k lenses for less than 1 year, and group II had been wearing ortho-k lenses for more than 1 year. The Pearson chi-square test (χ^2^) showed that the compliance rate of subjects wearing lenses for less than 1 year was higher than that of subjects wearing lenses for more than 1 year (*p* < 0.001) (Table 1). The compliance rate for each behavior in both groups is shown in Table 1. In group I, poorest compliance was with “lens case replacement according to ECPs’ recommendation” (55.8%) in addition to “removal of lens deposition interval according to ECPs’ recommendation” (67.3%) and “avoiding exposure of lenses to non-sterile solutions” (71.8%). In group II, poorest compliance was with “avoiding exposure of lenses to non-sterile solutions” (28%) in addition to “lens case replacement according to ECPs’ recommendation” (24.4%) and “removal of lens deposition interval according to ECPs’ recommendation” (39.0%). The Pearson chi-square test (χ^2^) showed that there was a significant difference in the compliance of the three wear and care behaviors between the two groups (Table 1).

To analyze the time period of compliance decline within 1 year, this study further analyzed the compliance rates in different periods within 1 year. The compliance rate for less than 1 month (*n* = 20), 1 month (*n* = 31), 3 months (*n* = 51), 6 months (*n* = 25), and more than 6 months (*n* = 29) of wearing experience was 45, 29, 21.6, 20, and 27.6%, respectively. The Pearson chi-square test (χ^2^) was used for multiple comparisons, and the results showed that there was no statistically significant difference among these periods (*p* = 0.314).

## Discussion

This study demonstrated that the compliance rate of ortho-k patients was low (19.7%), which is consistent with a study by Jiang et al. [18]. As with previous studies [18, 20], this study found that most ortho-k patients were teenagers in mainland China.

There was no correlation between compliance and lens care operator. The study also showed no correlation between compliance and age. Jiang et al. [18] surveyed the relationship between compliance and age in patients who had been wearing ortho-k lenses for more than 1 year, and the result was the same. Sapkota K also found that the compliance of conventional contact lens wearers was not related to age [14]. However, a study by Morgan PB et al. showed that the older the patient, the lower the compliance [16]. The different results may be due to the different ages of the subjects in these studies. The subjects in the study by Morgan PB had a large age span (range 21–60 years), while the subjects in other studies were of a certain age group.

No correlation has been found between compliance and sex. This result is the same as that of Jiang et al.’s study [18]. Conversely, some previous studies have shown that compliance in females is better than that in males [16, 21]. However, none of the subjects in those studies was ortho-k patients.

The study found that there was a correlation between wearing experience and the compliance with wear and care experience. The compliance of ortho-k patients wearing lenses for less than 1 year was significantly higher than that of patients wearing lenses for more than 1 year. A previous study by Jiang et al. showed that when ortho-k patients had worn lenses for more than 1 year, the compliance for wear and care behaviors had no correlation with wearing experience [18]. This result suggests that once ortho-k patients had worn lenses for 1 year, measures should have been taken to prevent compliance from decreasing. This result is consistent with a study by Wu et al. [22]; although they found that wearing experience was correlated with wear and care compliance for contact lenses other than ortho-k, compliance declined rapidly over the first 2 years rather than the first year. The following two reasons may be related to the faster decline of compliance among ortho-k patients. First, most ortho-k patients were teenagers in this study and were, therefore, younger than the patients in Wu et al.’s study (range 18–69 years; mean ± SD, 32 ± 1 years). Second, Wu et al.’s study investigated fewer behaviors than we investigated. Efron found that simpler guidelines may improve patient adherence [23].

The study investigated compliance over different periods in the first year and found no significant difference among them. The compliance in different periods in the first year was low, which should be noticed. It suggests that compliance with wear and care behaviors is poor at the beginning. However, 96.6% of subjects thought that their wear and care behaviors were correct. The reason for this strong contrast may be the same as the result of a study by Claydon [19]. The study showed that most non-compliant behaviors were not intentional and may have been due to the patient not remembering or understanding when ECPs illustrated the guidelines. However, none of the subjects in the study were ortho-k patients. Further studies are needed to verify this.

As shown in Table 1, during the first year, poorest compliance was with “lens case replacement according to ECPs’ recommendation” and “removal of lens deposition interval according to ECPs’ recommendation”. After wearing lenses for 1 year, compliance with these two behaviors remained poor, but compliance with “avoiding exposure of lenses to non-sterile solutions” decreased dramatically. This finding indicated that, while some non-compliance was unintentional, some patients were intentionally non-compliant. Therefore, it is important to re-examine ortho-k lenses patients’ wear and care practices to identify the wear and care behaviors that wearers are not following. A study by Jiang et al. [18] showed that after wearing ortho-k lenses for 1 year, compliance with “removal of lens deposition interval according to ECPs’ recommendation” and “avoiding exposure of lenses to non-sterile solutions” was poor, which is consistent with this study. However, compliance with “lens case replacement according to ECPs’ recommendation” in this study was much lower than that in the study by Jiang et al. It indicated that the behaviors with poor compliance might differ in different areas. Cho P et al. found that poor compliance behaviors could be improved by repeated education [17]. Therefore, the behaviors with poor compliance should be determined in different areas to re-educate the patients in those specific behaviors.

One of the limitations of this study is that the specific reasons for the decline in compliance are unclear; although it has been found that the compliance of ortho-k patients will decline with wearing experience. In addition, although repeated education has been shown to improve compliance [17], there is a need for further studies that would investigate the other effective strategies. It will be interesting to know whether different interventional measures can prevent and improve the compliance of ortho-k patients.

## Conclusions

In conclusion, our study confirmed that the compliance of ortho-k patients was poor. The duration of ortho-k wearing has been shown to influence compliance with wear and care behaviors. Therefore, ECPs should develop more effective ways of educating patients on the guidelines to improve compliance and take measures to prevent the reduction of compliance during wearing of ortho-k lens, especially after wearing ortho-k lenses for 1 year.

## Supplementary Information

**Additional file 1.**

## Data Availability

The datasets generated and/or analyzed during the current study are not publicly available due to the risk of individuals privacy being compromised but are available from the corresponding author on reasonable request.

## Abbreviations

ortho-k: Orthokeratology
RGP: Rigid gas-permeable
ECPs: Eye care professionals
ECSAHZU: Eye Center of the Second Affiliated Hospital of Zhejiang University School of Medicine
