# Supplementary material for: Assessment of patient compliance in orthokeratology and analysis of influencing factors: a cross-sectional study
Source: BMC Ophthalmol. 2021 Nov 16;21:396. doi: 10.1186/s12886-021-02148-2 (PMC8594163; doi:10.1186/s12886-021-02148-2)
Supplement: Supplementary file 1 — Additional file 1. [file 12886_2021_2148_MOESM1_ESM.docx]

**Additional file 1**

***1. Please indicate your information.***

Name: _________

Gender: _________

E-mail (optional): _____________

***2. Who is helping you wear your lenses*?**

A. Yourself B. Your parents C. Other________

***3. Who is helping you take off your lenses?***

A. Yourself B. Your parents C. Other________

*The following questions (4–13) should be answered by the doer.*

***4. Do you think your wear and care behaviors follow the eye care professionals’ guidelines?***

A. Always B. Sometimes C. Never

***5. Do you wash your hands before wearing your lenses?***

A. Always B. Sometimes C. Never

***6. Do you wash your hands before taking off your lenses?***

A. Always B. Sometimes C. Never

***7. Do you use soap when you wash your hands?***

A. Always B. Sometimes C. Never

***8. Do you dry your hands after washing your hands with tap water?***

A. Always B. Sometimes C. Never

***9. Do you wash your lenses before wearing your lenses?***

A. Always B. Sometimes C. Never

***10. Do you wash your lenses after wearing your lenses?***

A. Always B. Sometimes C. Never

***11. What kind of solution do you use to clean your lenses? (multiple-choice***)

A. Multipurpose contact lens solution B. Sterile water for injection

C. Tap water D. Boiled water E. Other

***12. Do you rub and rinse your lenses when washing your lenses?***

A. Always B. Sometimes C. Never

***13. How often do you replace the lens storage case solution?***

A. Each day when using lenses B. 2-7 days C. > 7 days

***14. How do you replace the lens storage case solution?***

A. Pour out used solution, then pour in fresh solution B. Topping off used solution C. Both of the above

***15. How often do you replace lens case?***

A. ≤ 3 months B. 3-6 months C. > 6 months

***16. How often do you provide intensive care for your lenses (removing lens protein deposition)?***

A. ≤ 2 weeks B. 2-4 weeks C. > 4 weeks

***17. How long do you use contact lens solution after it is opened?***

A. ≤ 3 months B. > 3 months
